# Supplementary material for: Ancestry-specific predisposing germline variants in cancer
Source: Genome Med. 2020 May 29;12:51. doi: 10.1186/s13073-020-00744-3 (PMC7260738; doi:10.1186/s13073-020-00744-3)
Supplement: Supplementary file 1 — Additional file 1: Figure S1. Principal component analyses (PCA) of germline TCGA samples to infer genetic ancestry as performed by PanCanAtlas Ancestry Informative Markers (AIM) working group. Figure S2. Power analysis for ancestry-specific sample sizes to discover predisposing genes. Figure S3. Nonsense-mediated decay prediction for predisposing frameshift variants in African and East Asian ancestries. [file 13073_2020_744_MOESM1_ESM.pdf]

UCSF PCA

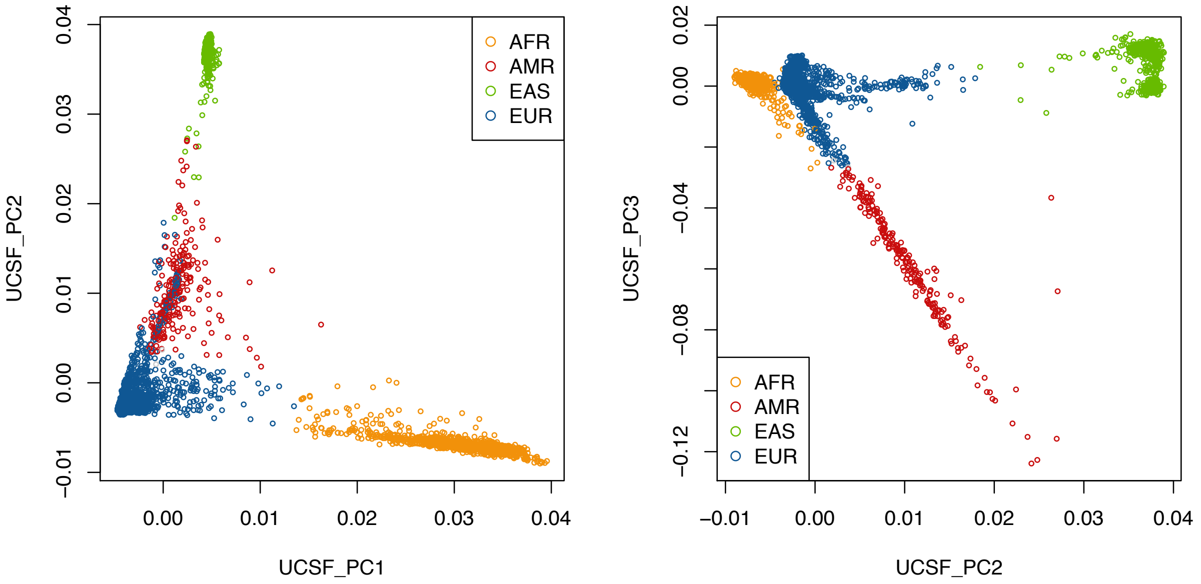

WashU PCA

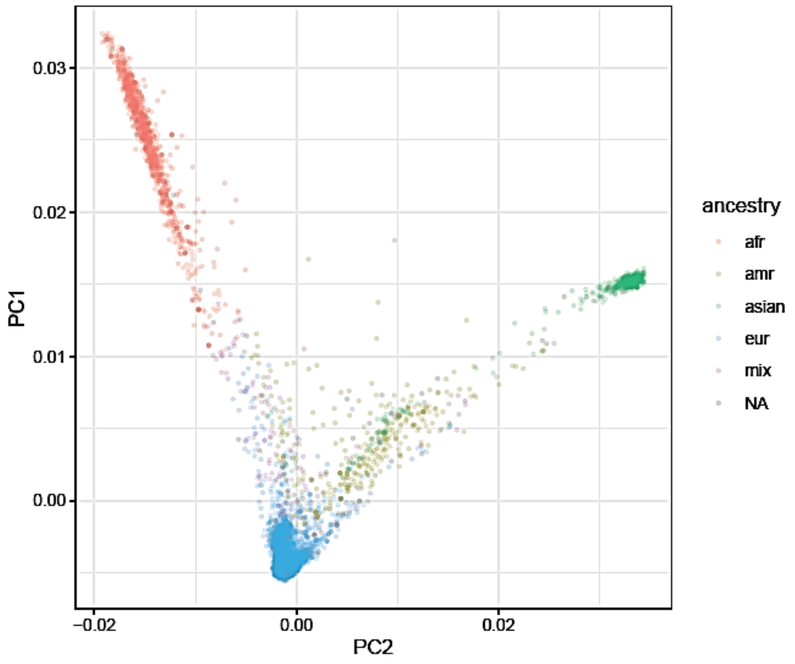

Fig S1 Principal component analyses (PCA) of germline TCGA samples to infer genetic ancestry as performed by PanCanAtlas Ancestry Informative Markers (AIM) working group

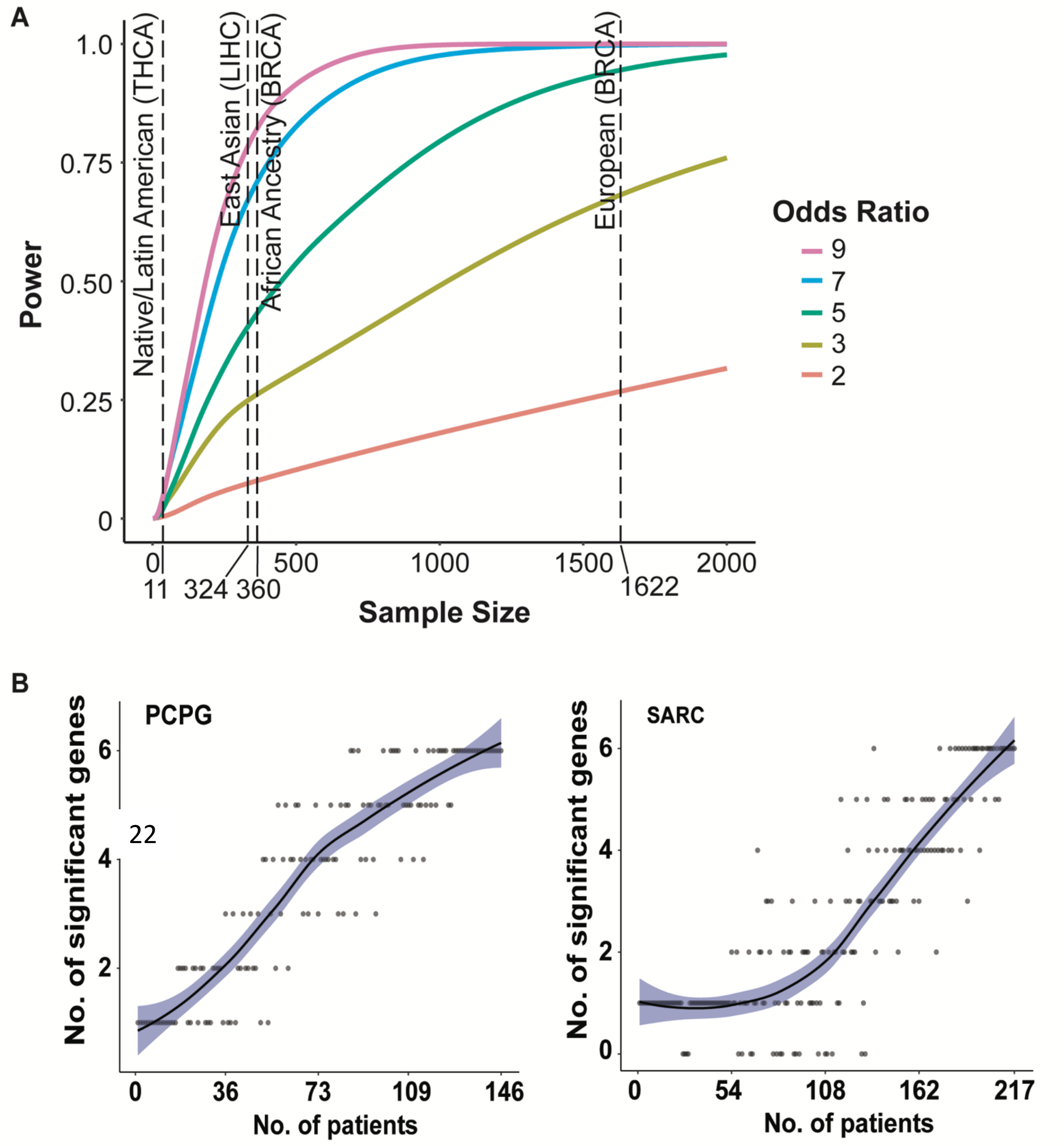

Fig S2 Power analysis for ancestry-specific sample sizes to discover predisposing genes.

A. The number of samples required to detect rare variant associations with varying effect sizes (OR = 2,3,5,7,9) at corresponding statistical power. The total number of samples assumes an equal number of cases and controls (e.g. For 811 TCGA-BRCA-EUR samples, n=1,622). Cancer types with the largest cohort sizes for each of the studied ancestries in TCGA are shown by a dotted line.

B. Down-sampling analysis to identify counts of significantly associated predisposing genes at different sample sizes by incrementally increasing the sample size from zero to the current cohort sizes.

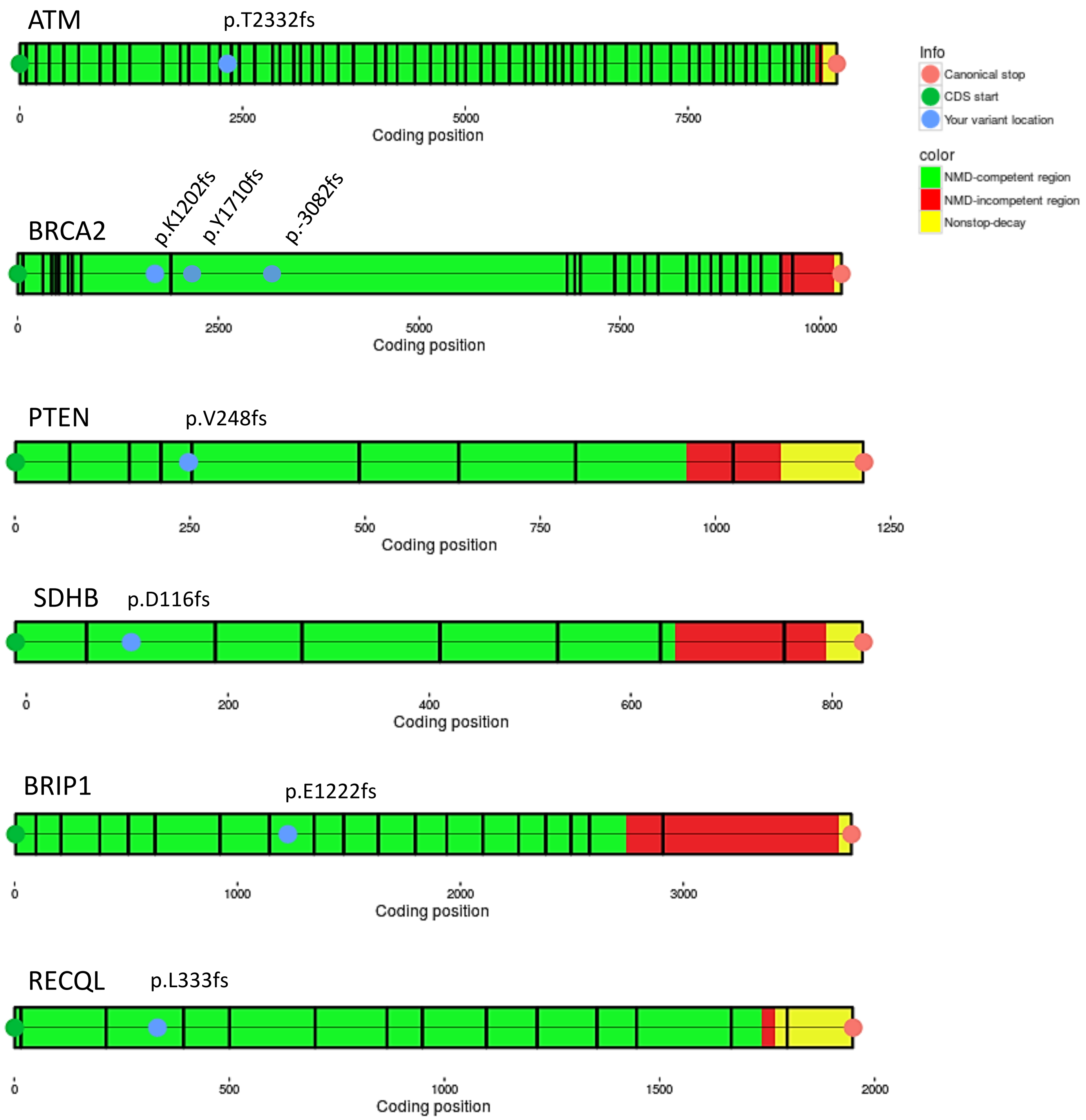

Fig S3 Nonsense-mediated decay prediction for predisposing frameshift variants in African and East Asian ancestries.
